# Treatment of Dyspepsia

**Published:** 1879-04

**Authors:** 


TREATMENT OF DYSPEPSIA.—According to the *Medical Record*, the following is the treatment of dyspepsia in the hospital of the University of Pennsylvania, at Philadelphia:

Among drugs, arsenic, in small doses, gradually increased, is a remedy of extreme importance. Where there is torpor of digestion joined with very marked sympathetic nervous disturbances, the following prescriptions are of great value:

| | | | |
|---|---|---|---|
| (1.) | ℞ | Sodæ bicarb. . . . . . . . . . . | ℨiij. |
| | | Acid hydrocyan. dil. . . . . | gtt. xlviij. |
| | | Tinct. valeriani. . . . . . . . . | f℥i. |
| | | Syrup. zingiberis. . . . . . . . | f℥ij. M. |

Sig. A teaspoonful thrice daily, in water.

| | | | |
|---|---|---|---|
| (2.) | R | Quiniæ sulph. . . . . . . . . | gr. xvi. |
| | | Strychniæ sulph. . . . . . . . | gr. ⅓. |
| | | Acid. muriat. dil. . . . . . . . | fℨiss. |
| | | Syrup. zingiberis. . . . | q. s. ad. f℥iv. M. |

Sig. Two teaspoonsfuls in water, right after meals.

Where there is marked hepatic disturbance, the following prescriptions are excellent:

| | | | |
|---|---|---|---|
| (3.) | R | Acid. muriat. dil. . . . . . . . | fℨss. |
| | | Tinct. nuc. vom. . . . . . . . . | fℨss. |
| | | Comp. infus. gentianæ . . . | q. s. ad. f℥iv. M. |

Sig. A teaspoonful in water after meals.

| | | | |
|---|---|---|---|
| (4.) | R | Bismuth. subnit. . . . . . . . . | ℨiss. |
| | | Pepsinæ. . . . . . . . . . . . | ℨiss. |
| | | Strichniæ sulph. . . . . . . . . | gr. i. |
| | | Tinct. cardamom. comp. . . | q. s, ad. f℥iv. M. |

Sig. A teaspoonful thrice daily, in water. If there is much flatulence, an increase is made in the quantity of bismuth and pepsin. If the case be merely one of gastric atony, the amount of strychnia is increased.

Where there is marked gastralgia, two to five drops of Fowler's solution are administered during the paroxysms. If this does not control the pain, a blister two inches square is applied to the epigastrium, and followed by a belladonna plaster six inches square.

Where the stomach is weak, its muscular action impaired, and its nerves over-sensitive, but little food should be taken into it at a time. The best diet is skimmed milk, half a pint every four hours. When milk is not well digested, lime-water is combined with it. Such foods as coffee, tea, and tobacco must, of course, be given up absolutely and at once. A sovereign article of diet is buttermilk. In buttermilk the casein of milk is coagulated and broken up, so that the stomach is spared two steps of the regular process of digestion. Another excellent preparation of milk is koumyss. It contains a good deal of carbonic acid. In all cases the stomach's work should be made easier by a diet consisting of eggs, milk, starchy vegetables, stewed fruits, and a little butter, with stale bread.—*Boston Jour. of Chemistry.*

